# Methods and challenges for the health impact assessment of vaccination programs in Latin America

**DOI:** 10.1590/S0034-8910.2015049006058

**Published:** 2015-12-16

**Authors:** Ana Marli Christovam Sartori, Andréia de Fátima Nascimento, Tânia Yuka Yuba, Patrícia Coelho de Soárez, Hillegonda Maria Dutilh Novaes

**Affiliations:** I Departamento de Moléstias Infecciosas e Parasitárias. Faculdade de Medicina. Universidade de São Paulo. São Paulo, SP, Brasil; II Departamento de Medicina Social. Faculdade de Ciências Médicas. Santa Casa de São Paulo. São Paulo, SP, Brasil; III Departamento de Medicina Preventiva. Faculdade de Medicina. Universidade de São Paulo. São Paulo, SP, Brasil

**Keywords:** Health Impact Assessment, Immunization Programs, Mass Vaccination, organization & administration, Rotavirus Vaccines, Pneumococcal Vaccines

## Abstract

**OBJECTIVE:**

To describe methods and challenges faced in the health impact assessment of vaccination programs, focusing on the pneumococcal conjugate and rotavirus vaccines in Latin America and the Caribbean.

**METHODS:**

For this narrative review, we searched for the terms “rotavirus”, “pneumococcal”, “conjugate vaccine”, “vaccination”, “program”, and “impact” in the databases Medline and LILACS. The search was extended to the grey literature in Google Scholar. No limits were defined for publication year. Original articles on the health impact assessment of pneumococcal and rotavirus vaccination programs in Latin America and the Caribbean in English, Spanish or Portuguese were included.

**RESULTS:**

We identified 207 articles. After removing duplicates and assessing eligibility, we reviewed 33 studies, 25 focusing on rotavirus and eight on pneumococcal vaccination programs. The most frequent studies were ecological, with time series analysis or comparing pre- and post-vaccination periods. The main data sources were: health information systems; population-, sentinel- or laboratory-based surveillance systems; statistics reports; and medical records from one or few health care services. Few studies used primary data. Hospitalization and death were the main outcomes assessed.

**CONCLUSIONS:**

Over the last years, a significant number of health impact assessments of pneumococcal and rotavirus vaccination programs have been conducted in Latin America and the Caribbean. These studies were carried out few years after the programs were implemented, meet the basic methodological requirements and suggest positive health impact. Future assessments should consider methodological issues and challenges arisen in these first studies conducted in the region.

## RESUMO

**OBJETIVO:**

Descrever métodos e desafios enfrentados na avaliação do impacto de programas de vacinação, com foco nas vacinas pneumocócica conjugada e de rotavírus nos países da América Latina e Caribe.

**MÉTODOS:**

Para esta revisão narrativa, foi realizada busca nas bases de dados Medline e Lilacs, usando os termos “rotavirus”, “pneumococcal”, “conjugate vaccine”, “vaccination”, “program”, e “impact”. A busca foi estendida à literatura cinza no Google Acadêmico. Não houve limitação por ano de publicação. Foram incluídos estudos originais de avaliação do impacto dos programas de vacinação de pneumococo e rotavírus na América Latina e Caribe, publicados em inglês, espanhol ou português.

**RESULTADOS:**

Foram identificados 207 artigos. Após remover duplicatas e avaliar elegibilidade, 33 estudos foram revisados, 25 sobre programas de vacinação de rotavírus e oito de pneumococo. Estudos ecológicos, com análise de série temporal ou comparação de períodos antes e após a vacinação, foram mais frequentes. As principais fontes de dados foram: sistemas de informação em saúde; sistemas de vigilância de base populacional, sentinela ou laboratorial; relatórios estatísticos; e prontuários médicos de um ou poucos serviços de saúde. Poucos estudos utilizaram dados primários. Hospitalizações e mortes foram os principais desfechos avaliados.

**CONCLUSÕES:**

Nos últimos anos, número significativo de avaliações do impacto dos programas de vacinação de pneumococo e rotavírus foram realizados nos países da América Latina e Caribe. Esses estudos foram conduzidos poucos anos após a implementação dos programas, preenchem os requisitos metodológicos básicos e sugerem impacto positivo dos programas. Futuras avaliações devem considerar questões metodológicas e desafios que surgiram nesses primeiros estudos conduzidos na região.

## INTRODUCTION

In the last thirty years, scientific and technological advances have resulted in the development and marketing of several new vaccines, increasing the opportunities to prevent morbidity and mortality related to infectious diseases of public health importance.^55^ In the 2000s, global and regional initiatives and commitment to immunization reduced prices of these new vaccines, which became accessible for low- and middle-income countries. Consequently, national immunization programs have been offering new vaccines.^55^ For instance, Brazil has provided eight new vaccines in the last eight years: rotavirus, in 2006; 10-valent pneumococcal conjugate and meningococcal C conjugate, in 2010; inactivated polio vaccine (IPV), in 2012; varicella, in 2013; and hepatitis A, in 2014, all of those in childhood schedule; in 2014, the human papilloma virus (HPV) vaccine, for teenage girls, and the tetanus-diphtheria-acellular pertussis (Tdap) vaccine, for pregnant women, were also introduced in 2014.

Once a new vaccine is introduced into routine immunization, it is necessary to monitor vaccine coverage, vaccine effectiveness and safety as well as the health impact of the vaccination program. Country differences in burden of disease, serotype and genotype distribution, health services organization and access, clinical practices, and surveillance systems prevent the use of international evidence as a guarantee of good results after implementing a program. Furthermore, vaccination programs may result in complex effects, changing the average age of infection, seasonal patterns of disease and genotype or serotype distribution.

Published studies use conflicting terms to describe different types of effects.^24^ Vaccine effectiveness is defined as the ability of a vaccine to protect against disease when used under field conditions (routine practice).^24^ Vaccine effectiveness refers to the protection conferred by individual immunization on vaccinated persons.^24^ Vaccination programs affect all people, even if only part of the population is vaccinated. When many people are immunized, the pathogen transmission decreases, which reduces the disease incidence and, consequently, protects the unvaccinated ones (indirect effect or herd protection). The health impact of a vaccination program refers to the total effects of the program, meaning the total (direct and indirect) effect on the vaccinees and the indirect effect on unvaccinated persons.^24^

Real-life effects of a vaccine administered in a health program are mainly evaluated in observational studies as experimental designs are no longer ethical once the vaccine is part of a health policy. Vaccine effectiveness may be estimated by comparing vaccinated and unvaccinated persons from the same population in cohort or case-control studies. The health impact of a vaccination program is estimated by comparing all individuals of the population affected by the vaccination program with a reference population unaffected by any program, usually the same population before and after program implementation. Different methodological approaches of differing complexity may be used.^24^

Countries with national health information systems, academic expertise in health services research, disease burden measurement and technology assessment in health care, policy makers, epidemiological surveillance, and immunization program professionals with experience in vaccine evaluations might have a more favorable context to conduct a health impact assessment (HIA) of vaccination programs.^51^ Nevertheless, introducing a new vaccine may be the opportunity for countries to create conditions for this evaluation, and others to follow, particularly if international organizations stimulate and support these initiatives.

The World Health Organization recommended that all national immunization programs offer rotavirus vaccine and pneumococcal conjugate vaccine (PCV),^56,57^ which have been introduced in Latin America and the Caribbean (LAC) from 2006 and 2008, respectively (Table 1). Hence, LAC countries have already had time to conduct HIA of rotavirus and pneumococcal vaccination.

**Table 1:** Pneumococcal conjugate and rotavirus vaccine introduction status and the health impact assessment of the vaccination program in the countries of Latin America and the Caribbean.

Country	Vaccine introduction status or year of introduction^a^		Health impact assessment of the vaccination program	

	Pneumococcal	Rotavirus	Pneumococcal	Rotavirus
Latin America				
Argentina	2012	No decision		
Belize	Planning introduction	No decision		
Bolivia	2014	2008		^11^
Brazil	2010	2006	^1,50^	^2,8,9,10,13,17,21,22,29,33,39,45,48^
Chile	2011	No decision		
Colombia	2011	2009	^38^	
Costa Rica	2008	Planning introduction		
Ecuador	2010	2007		
El Salvador	2010	2006		^11,59^
Guatemala	2012	2010		
Guyana	2011	2010		
Honduras	2011	2009		^11^
Mexico	2009	2007		^14,15,20,30,43,44^
Nicaragua	2010	2006	^7^	^37^
Panama	2010	2006	^35^	^6,32,34^
Paraguay	2012	2010		
Peru	2009	2009		
Suriname	No decision	No decision		
Uruguay	2008	No decision	^25,26,41,42^	
Venezuela	No decision	2006		^11^
The Caribbean^b^				
Bahamas	GAVI approved	GAVI plan		
Barbados	2009	No decision		
Cayman Islands		2009		
Cuba	Planning introduction	No decision		
Dominica	2010	No decision		
Dominican Republic	2009	2012		
Haiti	GAVI approved	2013		
Jamaica	2010	No decision		
Trinidad and Tobago	2009	No decision		

^a^ Source: International Vaccine Access Center – Vaccine Information Management System Report: Global Vaccine Introduction, March 2014.^27^

^b^ Caribbean countries that have not introduced pneumococcal nor rotavirus vaccines in their national immunization programs: Anguilla, Aruba, Antigua and Barbuda, French Guyana, Guadeloupe, Grenada, Martinique, Montserrat, Netherlands Antilles, Puerto Rico, Saint Kitts and Nevis, Saint Lucia, Saint Vincent and the Grenadines, Turks and Caicos Islands, Virgin Islands (UK and USA).

The objective of this study was to describe the methodological approaches (study design, data sources and outcomes of interest) used and the challenges to conduct HIA of PCV and rotavirus vaccination programs, with focus on LAC countries.

## METHODS

This is a narrative literature review of HIA of PCV and rotavirus vaccination programs, with focus on LAC countries. A search in Medline and LILACS, using the terms “rotavirus”, “pneumococcal”, “conjugate vaccine”, “vaccination”, “program”, and “impact”, was conducted on June 10, 2013 (PCV) and September 20, 2013 (rotavirus) and repeated on April 30, 2014. The review was supplemented with a search in Google Scholar to assess grey literature such as articles published in non-indexed journals, guidelines, and technical reports. There were no limits for publication year. Studies published in English, Spanish and Portuguese were eligible.

Three reviewers screened the identified abstracts and full texts and selected original articles that assessed the health impact of vaccination programs in LAC countries. Economic evaluations, mathematic models, vaccine efficacy or effectiveness studies and impact reviews were excluded. The references of all included articles were cross-checked and a hand search was carried out to identify further articles.

Data were extracted by one reviewer using a template developed specifically for this study and checked for accuracy by a second one. Data extracted from each study included: author, year, country, study design, data sources, clinical syndrome of interest, outcomes, and main results. Differences between reviewers were solved by discussion.

## ANALYSIS OF RESULTS

We initially identified 207 articles in the search: 92 on pneumococcal and 115 on rotavirus vaccination programs. After applying the exclusion criteria based on title and abstract reading, and checking for duplicates, we read 37 articles on PCV and 60 articles on rotavirus vaccine in full. The search update added seven articles to the set. Finally, we reviewed 33 studies on HIA of vaccination programs conducted in LAC, 25 of which assessed rotavirus and eight, PCV vaccination programs.

Of the 20 Latin American countries, 14 have introduced rotavirus vaccine in their immunization programs since 2006, and we identified at least one published HIA for eight of them (Table 1).^12,27^ In the Caribbean, only three of 25 countries have introduced rotavirus vaccine in their immunization programs since 2009 and no published HIA was identified. Since 2008, 17 Latin American countries have introduced PCV in their immunization programs and we identified at least one published HIA for five of them. Since 2009, five countries in the Caribbean have introduced PCV in their immunization programs and no published vaccination HIA was identified (Table 1).^12^ The 33 included LAC studies are described in Tables 2, 3 and 4 according to vaccine, data sources, country, and study design. In most LAC countries, rotavirus vaccines have been introduced earlier than PCV. Consequently, more HIA of rotavirus vaccination programs have been performed and published (25/33, 75.8%). Most studies were conducted in Brazil (15/33, 45.5%), mainly on rotavirus (13/25, 52.0%). Three of the eight studies on pneumococcal vaccine were conducted in Uruguay, one of the first LAC countries to implement a PCV childhood vaccination program.^25,26,41,42^

**Table 2:** Methodological characteristics of health impact assessments of rotavirus vaccination programs conducted in Latin American and Caribbean countries, based on secondary data (vital statistics, health services utilization or surveillance data).

Author/Year	Country	Study design/Methodological comments	Data source	Clinical syndrome	Outcome	Main results

Secondary data						
De Oliveira^11^ (2013)	Bolivia, El Salvador, Honduras, and Venezuela	Ecological (interrupted time-series analysis) Control: Argentina, which still had not introduced the vaccine into its national immunization program during the study period	Databases of the sentinel surveillance network of rotavirus diarrhea and records on hospitalizations and deaths from the ministries of health	All-cause diarrhea	Number of deaths and hospitalizations	Reductions in diarrhea-related deaths and hospitalization in all four countries as opposed to the control country
Rissardo^45^ (2010)	Brazil (Parana state)	Ecological (comparison of years before and after vaccine introduction) Limitations: short observation period after vaccine introduction; lack of adjustment for secular trends	National health information system on hospitalizations	All-cause diarrhea	Number and rates of hospitalizations	Significant decrease of diarrhea-related hospitalizations observed in children under one year of age after vaccine introduction. No impact evidenced among children aged one to four years
Do Carmo^9^ (2011)	Brazil	Ecological (interrupted time-series analysis, comparing event rates after vaccine introduction with expected rates estimated from prevaccine years) Adjustment for secular and seasonal trends	National mortality information system; national hospital information system, which covers the public health system	All-cause diarrhea	Mortality and hospitalization rates	Decreased rates of under-five diarrhea-related mortality and hospital admissions in the first three years after vaccine introduction, with largest reduction among children under two years of age
Lanzieri^29^ (2011)	Brazil	Ecological (comparison of years before and after vaccine introduction) Limitations: lack of adjustment for secular trends	Mortality information system; live birth information system	All-cause diarrhea	Mortality rates	Decreasing rates of diarrhea-related deaths previous to vaccine introduction
Gurgel^22^ (2011)	Brazil	Ecological (comparison of trends before and after vaccine introduction) Limitations: lack of adjustment for secular trends	National hospital information system (public health system)	All-cause diarrhea	Hospitalizations and deaths	Reduction in hospitalizations preceded the vaccine introduction
Fernandes^17^ (2014)	Brazil (Sao Paulo state)	Ecological (comparison of years before and after vaccine introduction)	National health information system (public health system)	All-cause diarrhea	Hospitalization rates by the human development index of each municipality and diarrhea-related hospitalization costs	Decreased rates of hospitalizations among under-five children in all categories of municipal development, with greater decrease in the least developed areas. Seasonal blunting in diarrhea hospitalizations. Savings in hospitalization costs in all municipal categories
Esparza-Aguilar^14^ (2009)	Mexico	Ecological (comparison of years before and after vaccine introduction)	National health information systems on mortality and population	All-cause diarrhea	Number of deaths and cumulative death rates	Decrease in diarrhea-related deaths previous to vaccine introduction. Greater mean annual reduction after vaccine introduction
Richardson^44^ (2010)	Mexico	Ecological (comparison of years before and after vaccine introduction)	National health information system on mortality and population	All-cause diarrhea	Deaths	Decline in diarrhea-related deaths after vaccination
Quintanar-Solares^43^ (2011)	Mexico	Ecological (comparison of years before and after vaccine introduction) Control: all-cause hospitalization	National health information system	All-cause diarrhea	Number of hospital admissions	Reduction in diarrhea-related hospitalizations only among children under 12 months of age in the first year after vaccine introduction, and among children under 24 months of age in the second year
Gastañaduy^20^ (2013)	Mexico	Ecological (comparison of years before and after vaccine introduction)	National health informatics system Data were classified into three regions, according to indicators of economic development	All-cause diarrhea	Deaths according to regional human development index	Reduction in diarrhea-related mortality sustained for four years after vaccine introduction. Comparable declines across the three regions of different levels of development
Esparza-Aguilar^15^ (2014)	Mexico	Ecological (comparison of years before and after vaccine introduction) Control: all-cause hospitalization	National health informatics system	All-cause diarrhea	Hospitalization rates according to the human development index of the state	Reduction in diarrhea-related hospitalizations of children under 24 months of age in all regions after vaccine introduction. Clear blunting of seasonal peaks
Nieto Guevara^34^ (2008)	Panama	Cross-sectional study (comparison of years before and after vaccine introduction) Limitations: short observation period	Statistics and medical records service database of one tertiary hospital	All-cause diarrhea	Number of hospitalizations; length of stay	No decrease in hospitalizations observed after vaccine introduction
Bayard^6^ (2012)	Panama	Ecological (comparison of years before and after vaccine introduction) Control: mean number of events in a prevaccination five-year period Limitations: short observation period	National mortality information system; hospital discharge database of five sentinel hospitals	All-cause diarrhea of presumed infectious origin	Mortality and hospital admissions	Decrease in diarrhea-related mortality and hospitalization rates after vaccine introduction
Yen^59^ (2011)	El Salvador	Ecological (comparison of years before and after vaccine introduction) Limitations: data on catchment population for the sentinel hospitals unavailable	Sentinel surveillance system (seven hospitals); national surveillance for diarrhea-related healthcare visits (inpatient and outpatient) in public healthcare facilities	All-cause diarrhea and rotavirus-positive diarrhea	Hospitalization rates and healthcare visits	Decreases in both hospitalizations and healthcare visits due to diarrhea
Orozco^37^ (2009)	Nicaragua	Ecological (comparison of years before and after vaccine introduction) Control: median number of events in a prevaccination four-year period. Limitations: Short observation period	National population-based surveillance for acute gastroenteritis events in healthcare facilities (Ministry of Health)	All-cause diarrhea	Number of outpatient visits and hospitalizations	Decreases in diarrhea-related hospitalizations and medical visits

Surveillance data						

Molto^32^ (2011)	Panama	Ecological (monthly trend analysis – comparison of years before and after vaccine introduction) Control: all-cause hospitalizations Limitations: unknown catchment population for the sentinel sites	National surveillance for diarrhea (six hospitals)	All-cause diarrhea	Hospitalizations	Reduction in diarrhea-related hospitalizations. Greater reduction during rotavirus seasonal months. All regions showed reduction in the ratio of diarrhea-related to non-diarrhea hospitalizations in the second year after vaccine introduction

Laboratory-based surveillance data						

Morillo^33^ (2010)	Brazil (Sao Paulo state)	Descriptive. Retrospective analyses of data collected in a five-year period, including two years before and two years after vaccine introduction	Laboratory-based data from diarrhea surveillance	Rotavirus-positive diarrhea	Proportion of rotavirus and rotavirus genotype distribution	Decrease in the proportion of rotavirus-positive samples before vaccine introduction. Emergence of the G2P[4] genotype after vaccine introduction
Carvalho-Costa^10^ (2011)	Brazil	Ecological	Laboratory-based surveillance: data from regional rotavirus reference laboratories in 18 of the 27 Brazilian federated units	Rotavirus-positive diarrhea, genotype characterization	Frequency of rotavirus and genotype distribution	Reduction in the proportion of rotavirus-related diarrhea in the years after vaccine introduction. Emergence of the G2P[4] genotype in the year before vaccination, with decrease in its detection in the last year of observation, probably reflecting natural genotype oscillations
Pereira^39^ (2011)	Brazil (Parana state)	Descriptive	Laboratory-based data from one tertiary hospital	Rotavirus-positive diarrhea	Proportion of rotavirus-positive samples	Decline in the proportion of rotavirus-positive cases two years before vaccine implementation
Dulgheroff^13^ (2012)	Brazil (Minas Gerais state)	Descriptive. Data from a four-year period after vaccine introduction were compared with prevaccination data from other studies conducted in the same region	Data from two laboratories that collect and analyze specimens from private and public hospitals and pediatric clinics from the region	Rotavirus-positive diarrhea and rotavirus genotype characterization	Proportion of rotavirus among hospitalized and outpatient acute gastroenteritis cases	Reduction in rotavirus-related diarrhea in comparison with prevaccination studies. Great reduction in genotype diversity with predominance of G2P[4]

**Table 3:** Methodological characteristics of health impact assessments of rotavirus vaccination programs conducted in Latin American and Caribbean countries, based on primary data collection.

Author/Year	Country	Study design	Data source	Clinical syndrome	Outcome	Main results
Primary data						

Gouvea^21^ (2009)	Brazil (Rio de Janeiro, RJ)	Hospital-based survey including three years and a half before and one year after vaccine introduction	Primary data collection at the emergency room of one hospital. Data gathered from medical bulletins and patient records. Vital statistics obtained from the Brazilian Ministry of Health	All-cause diarrhea and laboratory-confirmed rotavirus diarrhea	Number of emergency room visits, hospitalizations and deaths, and genotype distribution	The study was unable to clearly show the impact of vaccination. Gastroenteritis visits and hospitalizations showed significant year-to-year variation. A gradual decrease in rotavirus strain diversity was observed in the prevaccination years
Safadi^48^ (2010)	Brazil (Sao Paulo, SP)	Prospective cohort including years before and after vaccine introduction	Prospective primary data collection in a private hospital, with routine rotavirus testing for all under-five children hospitalized for acute gastroenteritis	All-cause diarrhea and rotavirus-positive diarrhea	Number of hospitalizations and genotype characterization	Reduction in the number of all-cause and rotavirus-related diarrhea hospitalizations; delay in the rotavirus seasonal peak; and predominance of G2P[4] genotype in the postvaccination period
Borges^8^ (2011)	Brazil (Goiania, GO)	Cross-sectional. Data collection restricted to the postvaccination period was compared with prevaccination studies conducted in the same region Limitations: small sample size, data collection in a period shorter than 1 year	Primary data collection in seven day care centers. Children were enrolled independent of gastrointestinal symptoms	Rotavirus-positive diarrhea, genotype characterization	Proportion of rotavirus-positive samples	Presence of rotavirus in 3.6% of all samples and 10.4% of samples from children with diarrhea were rotavirus-positive, which is less than what was previously observed by other studies in the region (14.4%-37.2%). G2P[4] was the predominant circulating genotype
Assis^2^ (2013)	Brazil (Juiz de Fora, MG)	Cross-sectional, including pre- and postvaccination years. Limitations: small sample size, compromising genotype distribution analyses. Data cannot be generalized to the entire Country	A university virology laboratory	Rotavirus-positive diarrhea	Frequency of rotavirus-diarrhea and genotype characterization	Decrease in the proportion of rotavirus-positive diarrhea after vaccine introduction. The G1P[6] genotype was most frequent before vaccine introduction and replaced by the G2P[4] genotype in the year of vaccine introduction and after
Leboreiro^30^ (2013)	Mexico	Case series. Retrospective (pre-vaccination) and prospective (post-vaccine introduction) analysis of a case series	Hospital medical records of children treated in one hospital, including the emergency room and hospital wards	All-cause diarrhea and rotavirus-positive diarrhea	Frequency and severity of diarrhea	Reduction in rotavirus-related diarrhea; reduction in rotavirus diarrhea severity among vaccinated children

**Table 4:** Methodological characteristics of health impact assessments of pneumococcal conjugate vaccination programs conducted in Latin American and Caribbean countries.

Author/Year	Country	Study design	Data source	Clinical syndrome	Outcome	Main results
Secondary data						

Afonso^1^ (2013)	Brazil (five capitals)	Ecological (interrupted time-series analysis) Control: non-respiratory causes Limitations: short observation period after vaccine introduction (one year)	National hospitalization information system (public health system). Data from five capitals that had good data quality and high PCV-10 vaccination coverage	All-cause pneumonia	Hospitalization rates among children aged two months to two years	Significant declines in the hospitalization rates for pneumonia in three capitals (Belo Horizonte, Curitiba and Recife), but not in the other two (Sao Paulo and Porto Alegre). Prevaccination hospitalization rates for pneumonia varied substantially by city. Hospitalization rates for non-respiratory causes also decreased in all cities, but at a lower rate
Nieto Guevara^35^ (2013)	Panama	Descriptive (retrospective), comparing a three-year period including pre- and postvaccination years. Involved indigenous population. Limitations: results cannot be generalized	Medical records of a secondary-level referral hospital, based on discharge diagnosis of pneumonia given by the treating physician and coded according to ICD	All-cause pneumonia	Hospitalization rates and transfers to the regional hospital among under-five children	Reduction in hospitalization rates and referrals for pneumonia were observed after vaccine introduction. Results cannot be generalized
Pírez^41^ (2011)	Uruguay	Descriptive (retrospective), comparing a three-year period before vaccine introduction with a one-year period after	A national tertiary referral pediatric hospital database, complemented by medical records, laboratory databases and reports from the national information system on notifiable diseases	All-cause pneumonia, pneumococcal pneumonia, pneumococcal meningitis	Hospitalization rates among children aged one month to 14 years	Reduction in hospitalization rates for pneumococcal pneumonia and pneumococcal meningitis after PCV-7 introduction. Non-vaccine serotypes 1, 5, 7F, 19A, and 24F became the most frequent causes of pneumococcal pneumonia after vaccine introduction. There were changes in the national hospital admissions aimed to decrease hospitalizations during the study. These changes did not affect the rates of hospital discharges for acute gastroenteritis, the control disease analyzed
Pirez^42^ (2014)	Uruguay	Descriptive (retrospective), comparing years before (2003-2007) and after (2009-2012) vaccine introduction	Microbiology laboratory database and patient records of a single site	All-cause pneumonia, pneumococcal pneumonia, pneumococcal serotypes	Hospitalization rates among children aged zero to 14 years	Significant reduction in hospitalization rates. A clear two-step reduction in hospitalization rates for pneumonia after each introduction of PCV (PCV-7 and PCV-13). Significant reduction in PCV-13 vaccine serotypes and increase in non-vaccine serotypes after the vaccination program implementation
Becker-Dreps^7^ (2014)	Nicaragua (León)	Descriptive, comparing a period before (2008-2010) and after (2011-2012) vaccine introduction. Control: healthcare visits due to diarrhea	Epidemiological database of 107 public health facilities (93 healthcare centers, 13 primary care centers, 1 public referral hospital)	Pneumonia	Hospitalization rates, outpatient visits among children aged 0 to 14 years and infant mortality	Reduction in hospitalization and outpatient visits for pneumonia and decrease in infant mortality after vaccine introduction. No changes in overall healthcare visits for diarrhea during the study period

Surveillance data						

Hortal^25^ (2007); Hortal^26^ (2012)	Uruguay	Two prospective cohorts – a three-year study before^27 ^and a three-year study after^28 ^vaccine introduction	Population-based surveillance system carried out in two municipalities. The study was conducted in four hospitals (two public and two private)	All-cause pneumonia	Annual hospitalization rates; serotype distribution among under-five children	Hospitalization rates for pneumonia did not decline in the three-year prevaccination study. Significant reduction in hospitalization rates for pneumonia and changes in serotypes after vaccine introduction

Primary data						

Santos^50^ (2013)	Brazil (Sao Paulo, SP)	Case series including years before and after vaccine introduction	Prospective data collection at one university hospital that attends a population of approximately 408,000 inhabitants	Invasive pneumococcal disease	Number of cases/1,000 hospital admissions; antibiotic resistance and serotype distribution	Decrease in invasive pneumococcal disease cases among children under 2 years of age and decrease in vaccine serotypes after vaccine introduction
Parra^38^ (2013)	Colombia	Case series on invasive pneumococcal disease and two transversal studies (before and after vaccine introduction) on nasopharyngeal carriage. Limitations: short observation period after vaccine introduction; children’s vaccination status unavailable	Laboratory surveillance (SIREVA II) data on invasive pneumococcal disease and primary data collection on nasopharyngeal carriage	Invasive pneumococcal disease and nasopharyngeal carriage serotype distribution	Serotype distribution	Decrease in vaccine serotypes and increase in non-vaccine serotypes in invasive pneumococcal disease isolates after vaccine introduction

ICD: International Classification of Diseases; PCV: pneumococcal conjugate vaccine; PCV-7: 7-valent pneumococcal conjugate vaccine; PCV-10: 10-valent pneumococcal conjugate vaccine; PCV-13: 13-valent pneumococcal conjugate vaccine

Ecological studies (interrupted time series analyses and other studies comparing pre- and postvaccination periods) were the most frequent (25/33, 75.7%). Cohorts (3), case series (3) and cross-sectional (2) studies were also conducted. Data sources were mainly secondary epidemiological or administrative databases (16/33, 48.5%). Surveillance data (8/33, 24.2%) and primary data collection (7/33, 21.2%) were also used. Two studies mixed data from both surveillance and health information systems.^6,11^ The study design was tied to data characteristics.

### Study design

Ecological studies are frequently used to evaluate epidemiological impact, especially when using large non-disease-specific databases, as they allow tracking population disease trends over time in relation to the timing of interventions. They allow both short- and long-term assessment of the vaccination program in a general population, but the establishment and measurement of causal relationship are limited because changes in disease incidence after vaccine introduction cannot be attributed exclusively to the intervention. Natural variations and secular trends affect disease incidences in the absence of vaccination.^11^ Changes in social and health conditions and improvement in access to healthcare system during the study may also influence the results of before-after studies.^11^ Strategies to control the effects of possible confounding factors include study design (comparison with other diseases or similar countries) and analyses (statistical methods to estimate the expected occurrence of the outcome using patterns before vaccine introduction, for example).

The rates of diarrhea-related hospitalizations and deaths of under-five children have been declining in LAC countries in the last three decades due to safe water supply, improvements in sanitation and hygiene, breastfeeding promotion, better nutrition, enhanced access to health care, and proper treatment of diarrhea, including oral rehydration therapy.^14^ This decline may be misinterpreted, overestimating the impact of vaccination. In some LAC studies, this decrease was already evident before vaccine introduction.^14,22,29^ Few studies adjusted for these secular trends appropriately in the analyses.^9,11,15,43^ A Brazilian study used a generalized linear model to compare the postvaccination years with expected rates estimated from prevaccination years adjusted for secular and seasonal trends.^9^ Two studies, in Mexico, used all-cause hospitalization to control these secular trends.^15,43^ A neighbor and similar country that had not implemented rotavirus vaccination was used as a control for possible secular trends in a HIA of rotavirus vaccination in four LAC countries.^11^

Rotavirus disease classically shows natural year-to-year variation, making it difficult to determine to which extent changes in disease trends are related to vaccination or to natural changes. Biennial increase in rotavirus activity has been reported in the postvaccine era.^55^ Unimmunized susceptible children accumulate during seasons with low rotavirus activity and the higher number of susceptible individuals facilitates transmission during a subsequent season.^53^ Temporal variability in rotavirus genotype distribution also occurs naturally, independent of vaccination.^4,10^ Proper assessment of vaccination impact requires monitoring for longer periods and careful interpretation.^10^

There is evidence of decreasing trends in pneumonia incidence and mortality in low- and middle-income countries from 2000 to 2010, attributed to economic and social developments, reduction in the prevalence of risk factors, expansion and improvement in case management and also the implementation of PCV and *Haemophilus influenzae* type b (Hib) childhood vaccination programs.^46^ Pneumonia also has a seasonal pattern and the observation period must last at least a year to consider these variations. Two LAC studies adjusted for possible secular trends in pneumonia rates using nonrespiratory and diarrhea events as controls (Table 4).^1,7^

### Data sources

Health information system databases were the main data source for HIA of vaccination programs in LAC. Thirteen studies assessed the impact of rotavirus vaccination based on health information systems, mainly mortality and hospitalization data at national level (Table 2).^6,9,11,14,15,17, 20,22,29,34,43-45^ One Brazilian study on the HIA of pneumococcal vaccination on hospitalizations used health information systems.^1^ The identification of hospitalizations or deaths in health information system databases relied on International Classification of Diseases (ICD) codes in discharge summaries or death certificates. Health information system databases are increasingly being used in research and health assessment. Some advantages are the broad coverage, lower costs of data collection, easy access to data, and the possibility of longitudinal follow-up. Major disadvantages are lack of standardization in data collection, which affects the quality of the information, time and space variation in coverage, and lack of important information for the analysis. There may also be delays on database availability. Changes in coding practices during the studied period may also affect the analyses.^9^ Failure to assign codes for gastroenteritis is a major reason for underestimating the burden of rotavirus disease and the impact of the vaccination program when using these data sources.^31^ Underdiagnosing or underreporting may also be an issue when estimating burden of disease and HIA of the vaccination program, leading to the development of models that use international data when adequate local data are unavailable.^51^

Important issues to be considered when using health information system databases are the completeness and reliability of the available data, coverage (proportion of population included), representativeness (whether persons included are similar to those not included), and sustainability (if the database is part of the health system and will be maintained long enough to monitor the effects of the program, independent of specific sponsoring). When using administrative databases, it is essential to consider the rules that govern the system and possible changes over time.^58^

In general, mortality databases are more reliable than morbidity or health services utilization databases because death is a single event and data are less affected by administrative or economic conditions. On the other hand, it measures only the effect on severe disease, and changes in less severe conditions will not be identified. Deaths occurring outside the health system, particularly in impoverished or rural areas, may not be registered in mortality systems.^6,44^ Additionally, undefined causes of death may constitute an important proportion of all deaths in developing countries. These factors might affect the analyses, particularly if changes occurred during the study period.^36^

Access to hospitalization information systems is increasing and they have been considered very useful as data sources in vaccination program impact analysis (Table 2).^9,15,17,44^ A study in Goiania, Midwestern Brazil, used database linkage of secondary administrative hospitalization data and primary population-based surveillance data and found similar hospitalization rates for community-acquired pneumonia in children.^50^

In many countries, burden of pneumococcal and rotavirus disease estimates are based on national sentinel-based surveillance data and the HIA of vaccination program relies on these data. Seven LAC studies evaluated the impact of rotavirus vaccination based on surveillance data (Table 2).^10,13,32,33,37,39,59^ Information on the catchment population of the sentinel hospitals is unavailable for most sites, precluding incidence rates estimation, which constitutes a limitation.^32,59^ Depending on the number of sentinel sites and their location, these data cannot be generalized for the entire population.^32,59^ The World Health Organization has proposed a sentinel surveillance system for rotavirus and invasive bacterial diseases,^a^ but some LAC countries established population-based surveillance for diarrhea with data collection for hospitalizations and outpatient visits at public health facilities. These population-based systems were used in HIA of rotavirus vaccination in Nicaragua and El Salvador, the latter in combination with sentinel hospital data.^37,59^ Population-based surveillance data were also used in HIA of a PCV program in Uruguay.^25,26^

The sensitivity of surveillance systems may change over time: variations in methods, case definitions, population under surveillance and reporting patterns may affect the results of before-after studies. In the era of PCV and rotavirus vaccines, most countries strengthened their surveillance systems to inform for decisions on vaccine policies.^54^ Furthermore, vaccine introduction increases disease awareness, testing and reporting.^23^

In LAC, invasive bacterial diseases surveillance was first organized as a laboratory-based surveillance system, the *Sistema de Redes de Vigilancia de los Agentes Bacterianos Responsables de Neumonia y Meningitis *(SIREVA II – Surveillance Network System for the Bacterial Agents Responsible for Pneumonia and Meningitis), created in 1993, initially in six countries (Argentina, Brazil, Chile, Colombia, Mexico, and Uruguay).^19^ The SIREVA II Regional 2012 Report contains data on pneumococcal serotypes and antibiotic resistance from 19 Latin American countries and the Caribbean Epidemiology Center.^b^ SIREVA II is a voluntary reporting system and its coverage varies a lot among countries, and caution is advised when using it to estimate invasive pneumococcal disease incidence and impact of PCV. In general, laboratory-based data lacks demographic and clinical information, which limits the analyses.^52^ Furthermore, laboratory procedures to identify the pathogen may change over time.^3,28^ Despite these limitations, SIREVA II is the best source of data on pneumococcal serotype distribution in the region and may allow assessing serotype replacement after vaccine introduction. Understanding serotype replacement is critical in low- and middle-income countries, where most deaths from pneumococcus occur, with greater diversity of serotypes causing disease and nasopharyngeal colonization early in infancy.^16^

Reports of laboratory-confirmed rotavirus infections from clinical microbiology laboratories that constitute a national- or sentinel-laboratory surveillance system were also used.^10,13,33,39^ These data allow assessing the impact of vaccination on rotavirus-confirmed diarrhea and genotype distribution.

Local secondary data including the hospital discharge summary database and medical records of a single hospital have been used as data sources for HIA of vaccination programs.^34,41,42^ The major limitation of these data is that study results cannot be generalized to the entire population.^30,35^

Primary data collection was conducted in HIA of PCV^50^ and rotavirus^2,8,21,30,48^ vaccination programs in LAC countries (Tables 3 and 4). Primary data collection may be particularly useful in settings where health information system databases are unavailable or unreliable and the surveillance system has not been appropriately implemented. Also, it can provide information unavailable on other data sources, such as rotavirus genotype circulation (Table 3).^2,48^ Limitations of studies based on primary data include the small sample size collected in just one or few sites, precluding generalizing the results to the whole population.^2,8,30^ Additionally, prospective design may be quite expensive, hampering the sustainability of the study and long-term HIA of the vaccination program.

### Study outcome definition

Choosing the clinical syndrome of interest in HIA of vaccination program is an issue. HIA of rotavirus vaccination used mainly all-cause diarrhea as the syndrome of interest and hospitalization or mortality rates as outcomes. Only two LAC studies that used population-based surveillance data assessed the impact of rotavirus vaccination on outpatient care (number of healthcare visits).^37,59^ The etiological diagnosis of rotavirus gastroenteritis requires laboratory tests, which are rarely performed in clinical practice since they do not alter the treatment.^17,59^ Rotavirus testing is done at the discretion of the physician, based on institutional practices, which may change over time.^53^ Although more specific and precise, using rotavirus-related diarrhea in studies based on secondary data may underestimate the true burden of disease and the impact of the vaccination program. Furthermore, “measuring impact on all-cause diarrhea may be more valuable to decision makers and the public health community because it provides an estimate of the preventable fraction of diarrhea deaths and admissions attributable to rotavirus”.^9^

Most of the eight HIA of PCV in LAC evaluated pneumonia,^1,7,25,26,35,41,42^ two evaluated invasive pneumococcal disease^38,50^ and one evaluated meningitis.^1^ We did not identify any LAC study evaluating the impact of PCV on acute otitis media.

Diagnosing invasive pneumococcal diseases require laboratory tests. In some countries, such as the USA, blood cultures (BC) are performed in routine care for every child with fever without a focus in both hospital and outpatient care, whereas in others, such as most LAC countries, BC are limited to severely ill hospitalized children. BC practices may affect the burden of disease estimates (invasive pneumococcal disease incidence increases parallel to the number of BC samples in a population), the relative frequency of clinical syndromes (higher frequency of bacteremia without focus in countries with higher frequency of BC samples) and the serotype distribution. Previous use of antibiotics before sample collection also affects diagnostic sensitivity.^40,54^ Changes in medical practices may also influence the results of before-after studies. A study of invasive pneumococcal disease before and after the PCV7 program implementation in England and Wales evaluated control pathogens that also depend on blood culture practices and reporting, but for which there had been no public health intervention (*Escherichia coli* and non-pyogenic streptococci).^18^ Similar trends (increasing rates) for invasive pneumococcal disease and the control pathogens suggested that the sensitivity of surveillance was increasing prior to vaccine introduction. Ignoring prevaccination trends could lead to underestimating the reduction in invasive pneumococcal disease and overestimating the degree of replacement disease.^18^

Technical developments may increase the sensitivity of diagnostic tests.^54^ Introduction of polymerase chain reaction (PCR) in the cerebral spinal fluid for diagnosing bacterial meningitis may increase the number of laboratory-confirmed pneumococcal meningitis cases.^47^ This must be considered when analyzing changes in diagnostic test results throughout the period evaluated.

Pneumonia definition is a challenge.^46^ Bacteremia occurs only in a small proportion of cases. The etiological diagnosis of non-bacteremic pneumonia by current tests is insufficiently sensitive and specific, and rarely performed in clinical practice. Due to difficulties to isolate the etiological agent, most studies focused their analyses in all-cause pneumonia.^1,25,26,35,41,42^ Some of them also evaluated pneumococcal pneumonia (PP).^41,42^ Definitions of pneumonia vary among studies. In general, studies based on secondary data used the diagnosis given by the attending physician, but diagnostic criteria vary among clinicians, health services and health information system databases.^35^ Prospective cohorts used more standardized criteria, mainly radiologically-confirmed pneumonia.^25,26^

Although the clinical diagnosis of acute otitis media does not require additional exams, and the collection of material to isolate pathogens is easier than for pneumonia, LAC countries lack high quality data on acute otitis media incidence and health resource use.^5^ Generally, acute otitis media is treated in outpatient services, for which registered information is limited in LAC, hindering the HIA of pneumococcal vaccination on this disease.

### Main results of the health impact assessment of rotavirus and pneumococcal vaccination programs in Latin America

Most studies showed decreased rates of diarrhea-related deaths, hospital admissions and healthcare visits after rotavirus vaccination implementation (Tables 2, 3 and 4). Blunting or delay of seasonal peaks of diarrheal disease after vaccine introduction has also been reported.^15,17,32,48^ Two studies in Mexico and another in Brazil assessed the impact of rotavirus vaccination on diarrhea mortality or hospitalization rates according to the socioeconomic level or human development index of the region.^15,17,20^ The Mexican studies observed comparable reduction in diarrhea-related deaths and hospitalization in all areas, whereas the Brazilian study showed great reduction in hospitalization rates of under-five children in the least developed areas.^15,17,20^

All eight HIA of pneumococcal vaccination programs in LAC showed reduction in the events of interest, mainly hospitalization, after PCV introduction (Table 4). Nonvaccine serotypes increased in Colombia and Uruguay after vaccine introduction.^38,41,42^

Table 5 presents a summary of advantages and limitations of study design, data sources, and outcomes used in HIA of vaccination programs.

**Table 5:** Summary of advantages and limitations of study design, data sources, and outcomes of interest used in HIA of vaccination programs.

	Advantages	Limitations
Study design		

Ecological	Relatively simple and less expensive Allows assessment of the vaccination program in a general population	Cannot establish causal relationships It is important to consider adjustment for secular trends and to have a control group (other areas without vaccination programs or other diseases)
Cohort	Provides the best information about causal relationships	Demands more time and resources
Descriptive and case series	Simplest study designs Can detect changes in types of rotavirus and pneumococcus after the introduction of vaccine programs	Cannot measure prevalence or incidence due to the lack of a well-defined population at risk. Give little information about temporal changes in the frequency of diseases

Data sources		

Health information system databases	Broad coverage, lower costs, easy access to data and longitudinal follow-up	Lack of standardization in data collection and limitations in completeness and reliability of the available data, database continuity, coverage, representativeness, and sustainability
Sentinel-based and laboratory-based surveillance systems	Availability of unpublished data	Information on the catchment population frequently unavailable. Data not generalizable for the entire population. Variable coverage
Local secondary data (medical records of a single hospital)	Timeliness, low costs, and availability	Lack of standardization; data not generalizable to the entire population
Primary data	May answer specific research objectives More precision and reliability	Small sample size Not generalizable Expensive Sustainability

Clinical syndrome		

All-cause diarrhea	Does not require specific laboratory tests. May be more valuable to decision makers	Less precision for vaccine effect on rotavirus disease
Rotavirus-related diarrhea	More specific and precise	May underestimate the true burden of disease and the global impact of vaccination programs
Pneumonia	More frequent	Challenging and variable definition and diagnosis
Invasive pneumococcal disease	More severe and of more precise diagnosis	Diagnosis requires the isolation of *S. pneumonia, *and laboratory tests are not uniformly performed
Meningitis	More severe, of more precise diagnosis and has more information available since it is a notifiable disease	Less frequent

Outcomes of interest		

Hospitalization rate	Availability of data	Changes in diagnosis coding may affect estimates. Influenced by availability of beds, hospital admission policies and social factors. Unsuitable for clinical syndromes that are mostly treated in outpatient care such as otitis media
Mortality rate	More reliable than morbidity data	Measures only results of severe clinical syndromes; thus, changes in less severe conditions will not be identified. Difficulty in discriminating effects of changes in the incidence or treatment of conditions

Despite the strategies to access grey literature, country reports and other local documents may not be included in this review. The classification of epidemiological study designs was heterogeneous and we used the authors’ classification. Furthermore, some studies lack methodological information. These two limitations may affect our analysis and synthesis of knowledge production on HIA of PCV and rotavirus vaccination programs.

The challenges in conducting HIA of vaccination programs are easier to face in countries with reliable and sustainable health information systems and surveillance data as well as expertise in health evaluation. However, LAC countries have managed to do a lot in HIA of pneumococcal and rotavirus vaccination programs in a relatively short time after program implementation. Almost all met basic methodological requirements for HIA and suggested a positive health impact. High-quality studies have been conducted in small countries without tradition in research that have prioritized surveillance and registers. Future HIA of vaccination programs should consider the methodological issues and challenges that arose in these first studies conducted in the region as well as in studies from other regions. HIA of vaccination programs should be considered essential in the planning phase of vaccine introduction, with the definition of outcomes, data sources, and responsibilities for data collection and resources. They can also contribute to the validation and methodological development of vaccine cost-effectiveness studies.

## Footnotes

a World Health Organization, Department of Immunization, Vaccines and Biologicals, Expanded Programme on Immunization. Summary report and action plan for 2012 from the Global Meeting on Surveillance for Vaccine Preventable Invasive Bacterial Diseases (VP-IBD) and Rotavirus; 22-24 Sept 2010; Geneva, Switzerland. Geneva: WHO; 2010 [cited 2014 May 4]. Available from: http://www.who.int/immunization/monitoring_surveillance/resources/Full_2010_Surv_Mtg_Report-21_Jan_2011.pdf

b Organizacion Panamericana de la Salud. Informe Regional de SIREVA II, 2012: datos por país y por grupos de edad sobre las características de los aislamientos de *Streptococcus pneumoniae, Haemophilus influenzae *y* Neisseria meningitidis* en procesos invasores. Washington (DC); 2013 [cited 2014 May 4]. Available from: http://www.paho.org/hq/index.php?option=com_content&view=article&id=5461:sireva-ii-sistema-de-redes-de-vigilancia-de-los-agentes-responsables-de-neumonias-y-meningitis-bacterianas-&Itemid=3953&lang=pt
